# Announcement

**Published:** 2015-05-08

## CDC-Sponsored Continuing Education Courses on Screening for Colorectal Cancer

Colorectal cancer is the second leading cancer killer in the United States. Screening for colorectal cancer saves lives, but problems with its implementation in clinical practice can reduce screening’s effectiveness.

A new CDC-sponsored continuing education program is available to provide guidance and tools for clinicians on the best ways to implement screening for colorectal cancer to ensure patients receive maximum benefit.

There are two versions of the course: one for primary care providers and one for clinicians who perform colonoscopy procedures. The courses were developed by nationally recognized experts in colorectal cancer screening, including primary care clinicians, gastroenterologists, and leaders in public health programs and research. Continuing education credits are available for physicians, nurses, and other health professionals.

The courses can be accessed free of charge at http://www.cdc.gov/cancer/colorectal/quality.

